# Metformin-Associated Lactic Acidosis following Intentional Overdose Successfully Treated with Tris-Hydroxymethyl Aminomethane and Renal Replacement Therapy

**DOI:** 10.1155/2012/671595

**Published:** 2012-05-23

**Authors:** Ngan Lam, Gurbir Sekhon, Andrew A. House

**Affiliations:** ^1^Division of Nephrology, Department of Medicine, Western University, London, ON, Canada N6A 3K7; ^2^London Health Sciences Centre, Kidney Clinical Research Unit, Victoria Hospital, Westminster Tower 800 Commissioners Road East, London, ON, Canada N6A 4G5; ^3^Department of Medicine, Western University, London, ON, Canada N6A 3K7

## Abstract

A 43-year-old woman was brought to the hospital with severe metabolic acidosis (pH 6.56, bicarbonate 3 mmol/L, and lactate 18.4 mmol/L) and a serum creatinine of 162 *μ*mol/L with a serum potassium of 7.8 mmol/L. A delayed diagnosis of metformin-associated lactic acidosis was made, and she was treated with tris-hydroxymethyl aminomethane (THAM) and renal replacement therapy (RRT). Following a complete recovery, she admitted to ingesting 180 tablets (90 grams) of metformin. Her peak serum metformin concentration was 170 *μ*g/mL (therapeutic range 1-2 *μ*g/mL). Our case demonstrates an intentional metformin overdose resulting in lactic acidosis in a nondiabetic patient who was successfully treated with THAM and RRT.

## 1. Background

Metformin is an oral antihyperglycemic agent that is the first-line therapy for noninsulin-dependent diabetes mellitus [1]. Although the adverse event rate is 20–30%, the majority of these are gastrointestinal symptoms such as abdominal pain and nausea [2]. Metformin has been associated with an increased risk of lactic acidosis through the inhibition of hepatic gluconeogenesis causing an increased level of lactate, pyruvate, and alanine. The incidence of metformin-associated lactic acidosis (MALA) is reported to be 3 per 100,000 person-years [3]. The symptoms of severe MALA can be nonspecific, such as tachycardia, hypotension, tachypnea, and mental status changes, potentially misleading the diagnosis and delaying treatment.

## 2. Case Report

A 43-year-old woman was brought to the emergency room complaining of chest pain for 20hours radiating to her back. Her medical history was significant for hypertension for which she was on atenolol 50 mg daily and anxiety for which she was on mirtazapine 30 mg daily and lorazepam 2 mg three times a day. 

Her temperature was 32.3°C (axilla), blood pressure 66/45 mmHg, heart rate 57 beats per minute, and oxygen saturation 97% on 3 liters. Her respiratory, cardiac, and abdominal exams were unremarkable. In addition to normal cardiac enzymes, her initial laboratory values revealed the following: serum creatinine 162 *μ*mol/L, potassium 5.3 mmol/L, bicarbonate 4 mmol/L, anion gap 31 mmol/L, lactate 18.4 mmol/L, osmolar gap 18.3 mOsm/kg, venous blood gas: pH 6.56, pCO2 35 mmHg, bicarbonate 3 mmol/L (Table 1). Four hours after she first arrived, her condition rapidly deteriorated, and she became obtunded requiring intubation and transfer to the intensive care unit (ICU). 

Prior to intubation, the patient adamantly denied any toxic ingestion. She was treated with levofloxacin and piperacillin/tazobactam for potential sepsis. Her acidosis was treated with sodium bicarbonate infusion. Passive external rewarming therapy was used to treat her hypothermia. Her hypotension required isotonic intravenous fluids and vasopressor agents including phenylephrine, norepinephrine, dobutamine, and vasopressin. Cardiovascular/thoracic surgery was consulted to rule out aortic dissection, and a transesophageal echocardiogram was inconclusive. The patient underwent an urgent CT thorax/abdomen/pelvis with contrast, which did not reveal an aortic dissection or abscess.

Biochemically, she continued to worsen with a peak serum potassium of 7.8 mmol/L treated with calcium chloride, calcium gluconate, and kayexalate. She became hypoglycemic with a nadir blood sugar of 1.2 mmol/L treated with dextrose intravenously. Her anion gap rose to 43 mmol/L and her peak osmolar gap was 29.3 mOsm/kg. Her urine toxicology screen was positive for opiates and benzodiazepines but negative for cannabis, cocaine, amphetamine, and barbiturates. Her serum acetaminophen level was 81 *μ*mol/L but the remainder of her serum toxicology screen was negative for tricyclics, salicylates, ethanol, methanol, isopropanol, acetone, and ethylene glycol.

The patient's hospital chart arrived to the ICU where it was discovered that the patient had a history of a suicide attempt by overdose 15 years prior. It was also discovered that a family member had diabetes treated with metformin. A diagnosis of metformin-associated lactic acidosis by intentional overdose was made.

Nephrology was consulted and a temporary internal jugular hemodialysis (HD) catheter was inserted. The patient was treated with 6 hours of HD starting 12 hours after her initial arrival to the hospital. She was also started on tris-hydroxymethyl aminomethane (THAM, 0.3 mmol/L, 36 mg/mL, 1600 mg administered by infusion at 300 mL/hr for 5 hours) to buffer the lactic acid given the preservation of her urine output, which was maintained at approximately 200 mL/hr. Given her initial level of acetaminophen and a rise in her liver enzymes, she was treated with N-acetylcysteine (NAC) for a possible missed acetaminophen overdose.

The patient received one 8-hour session of continuous veno-venous hemofiltration (CVVHF) the next day for ongoing metabolic acidosis. Her condition dramatically improved, and she was extubated the following day. Her renal function returned to baseline and she did not require any further renal replacement therapy (RRT). She admitted to taking 180 tablets (500 mg each, 90 grams total) of metformin. Her peak serum metformin level, taken almost 8 hours after her arrival to the hospital, was 170 *μ*g/mL (therapeutic range 1-2 *μ*g/mL). 

## 3. Discussion

A recent Cochrane review demonstrated that in patients with type 2 diabetes, who were using metformin therapeutically, there were no cases of fatal or nonfatal lactic acidosis nor was there an increased risk of elevated lactate compared with other anti-hyperglycemic agents [24]. This review did not apply to intentional metformin overdose for which the literature is limited and single case reports continue to be published (Table 2) [4–23]. Intentional overdoses involving healthy individuals with no history of diabetes are even more rare [4, 6, 10, 15, 17, 19, 21]. In the majority of cases, the diagnosis is obvious based on patient and collateral history, and treatment is initiated in a timely fashion.

The initial management of MALA is supportive and may include gastrointestinal decontamination if the ingestion was recent and no contraindications are present. Further treatment options include intravenous hydration, sodium bicarbonate, dextrose for hypoglycemia, intubation for airway protection, and mechanical ventilation for respiratory failure, vasopressor support and RRT. The role of RRT appears to be in the correction of the acidosis as opposed to the clearance of metformin, although some limited clearance can occur [20, 23]. Various forms of dialysis, either alone or in combination, have been used to treat MALA, including intermittent HD [4–9, 11–15, 17, 19, 21], hemoperfusion (HP) [12], CVVHF [9, 11, 13, 20], continuous veno-venous hemodialysis (CVVHD) [15, 22, 23], continuous veno-venous hemodiafiltration (CVVHDF) [5, 15, 16], and plasma exchange (PLEX) [8], all with varying outcomes. In some cases, RRT is not utilized if the degree of acidosis or acute kidney injury is not severe or the patient is not acutely ill [10, 18, 25].

The role of sodium bicarbonate in the treatment of MALA is widely debated and controversial. Sodium bicarbonate may deliver an unwanted sodium load resulting in hypervolemia. It is also a carbon dioxide producing buffer, which may decrease cardiac output [17]. THAM buffers acid without the production of excess carbon dioxide and is used as an alternative to sodium bicarbonate [26]. The hydrogenated THAM is then excreted in the urine making it less efficacious if the patient is anuric. The clinical efficacy of THAM compared with sodium bicarbonate in the treatment of severe metabolic acidosis is currently unproven.

To our knowledge, this is the first published case of MALA successfully treated with THAM and RRT. While dialysis is used in the majority of cases of MALA, we demonstrate that THAM may also have a role in alkalinization for the treatment of severe lactic acidosis associated with metformin overdose. As far as we know, our nondiabetic patient survived despite having the lowest documented pH at 6.56 associated with a high metformin concentration of 170 *μ*g/mL.

## Figures and Tables

**Table 1:** Laboratory investigations.

Results	Initial	4 hours after arrival	6 hours after arrival	10 hours after arrival (before RRT)	Final (after RRT)
Arterial pH	6.83	6.92	6.82	7.01	7.43
Arterial bicarbonate (mmol/L)	3	4	3	4	31
Arterial pCO_2_ (mmHg)	20	18	18	16	47
Sodium (mmol/L)	145	146	145	153	139
Potassium (mmol/L)	5.3	7.5	7.7	6.5	3.5
Chloride (mmol/L	110	114	110	107	104
Bicarbonate (mmol/L)	4	4	3	3	24
Anion gap (mmol/L)	31	28	32	43	11
Urea (mmol/L)	1.9	2	2.2	2.6	6.1
Serum creatinine (*μ*mol/L)	162	158	159	162	61
Albumin (g/L)	31	36	33	35	—
Plasma glucose (mmol/L)	7.8	1.2	8.5	3.9	8
Serum osmolality (mOsm/kg)	318	—	330	—	—
Osmolar gap (mOsm/kg)	18.3	—	29.3	—	—
Leukocytes (×10^9^/L)	26.8	27.3	34.1	28.7	11.9
Creatine kinase (U/L)	87	77	—	—	—
Troponin-T (*μ*g/L)	<0.01	<0.01	—	—	—
Alanine aminotransferase (U/L)	—	80	110	127	77
Aspartate aminotransferase (U/L)	—	77	108	124	62
Alkaline phosphatase (U/L)	—	71	69	65	66

pCO_2_: pressure of carbon dioxide, pH: potential hydrogen, RRT: renal replacement therapy.

**Table 2:** Case reports of intentional overdose resulting in metformin-associated lactic acidosis, in chronological order grouped by survival and death^a^.

Case	Age in years, sex	Diabetic yes or no	Amount grams	Peak metformin level *μ*g/mL	Nadir pH	Nadir HCO_3_ mmol/L	Peak lactate mmol/L	Peak creatinine *μ*mol/L	Glucose mmol/L^b^	RRT mode
					Survived					
Present case	43, F^c^	No	90	170	6.56	3	18.4	162	1.2	HDCVVHF
Rathnapala et al. [4]	18, F^c^	No	17.5	—	7.08	<3	60.0	>900	2.3	HD
Rifkin et al. [5]	50, M	Yes	52	10.2	6.84	3	21.2	371	—	HDCVVHDF
Akoglu et al. [6]	34, M	No	144.5	—	7.10	3.6	17.8	239	7.9	HD
Dell'Aglio et al. [7]	40, F	—	75–100	160	6.59	5	40.0	203	—	HD
Turkcuer et al. [8]	30, F	—	85	—	6.88	7.3	22.4	220	8.3	HDPLEX
Yang et al. [9]	43, F	Yes	—	—	6.99	4.7	39.3	716	0.9	HDCVVHF
Basturk et al. [10]	25, F	No	100	—	7.16	9.7	17.7	147	6.1	None
Galea et al. [11]	46, M^c^	Yes	56	—	6.80	3.9	>15	368	8.2	CVVHFHD
Guo et al. [12]	37, M^c^	Yes	45	—	6.81	4	>30.0	—	—	HDHD + HP
	53, M^c^	Yes	50	—	6.85	3	>30.0	—	—	HD
Panzer et al. [13]	42, M	Yes	—	191	6.89	6.7	25.8	159	—	HDCVVHF
Gjedde et al. [14]	70, M	Yes	63	—	7.04	9.2	23.6	216	0.4	HD
Chang et al. [15]	58, F^c^	Yes	55	—	6.80	1.8	5.5	194	5.2	HD
Teale et al. [16]	60, M^c^	Yes	50	—	6.9	4	32	526	7	CVVHDF
Heaney et al. [17]	29, M^c^	No	—	—	6.96	2	31	398	0.6	HD
McLelland [18]	83, F	Yes	25	42	6.98	4	25	—	14	None

					Died					
Perrone et al. [19]	40, F^c^	No	—	150	6.95	25	21	—	7.8	HD
Arroyo et al. [20]	49, F^c^	Yes	30	380	6.79	3.1	39.1	106	32.1	CVVHF
Suchard and Grotsky [21]	29, M^c^	No	64–85	—	6.95	6	>11.1	186	39.2 (peak)	HD
Nisse et al. [22]	42, M	Yes	—	188	6.88	2.9	27	163	16.4	CVVHD
Barrueto et al. [23]	58, M^c^	Yes	20	110	6.6	<5	33.3	221	—	CVVHD
Chang et al. [15]	25, F	No	—	—	7.10	4.4	33.2	141	10.9	CVVHD CVVHDF
Teale et al. [16]	25, M	Yes	—	—	6.79	2.1	25	—	0.3	CVVHDF
	33, F	Yes	35	—	6.7	5.1	—	—	2	None

^a^Reported values are shown.

^b^Unless otherwise stated, reported or nadir glucose values are shown.

^c^Represents reported polypharmacy overdose.

CVVHD: continuous veno-venous hemodialysis, CVVHDF: continuous veno-venous hemodiafiltration, CVVHF: continuous veno-venous hemofiltration, F: female, HCO_3_: bicarbonate, HD: hemodialysis, HP: hemoperfusion, M: male, pH: potential hydrogen, PLEX: plasma exchange, RRT: renal replacement therapy.
